# Malignant melanoma of parotid glands from a neglected lesion: A case report

**DOI:** 10.1002/ccr3.4941

**Published:** 2021-10-13

**Authors:** Mahsa Ahadi, Azadeh Rakhshan, Seyed Reza Mousavi, Homeira Saebnoori

**Affiliations:** ^1^ Department of Pathology Shohada_e_Tajrish Educational Hospital Shahid Beheshti University of Medical Sciences Tehran Iran; ^2^ Department of Pathology Skin Research Center Shahid Beheshti University of Medical Sciences Tehran Iran; ^3^ Department of Surgery Shohada_e_Tajrish Educational Hospital Shahid Beheshti University of Medical Sciences Tehran Iran; ^4^ Department of oral and maxillofacial pathology Tehran University of Medical sciences, School of Dentistry Tehran Iran

**Keywords:** immunohistochemical staining, malignant melanoma, metastasis, parotid glands, scalp skin

## Abstract

Observing a metastatic malignant melanoma and its primary lesion at the same time is rare. The histopathological detection of any unusual pigmented lesion is critical.

## Graphical abstract

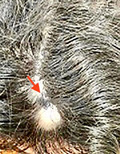

## 1 INTRODUCTION

This is a report of a patient with metastatic melanoma of bilateral parotid glands derived from a neglected pigmented lesion of the scalp. Any unusual pigmented lesion after surgical removal should be microscopically investigated. In the case of metastatic melanoma of parotid with no specific primary origin, patient’s history is helpful. Malignant melanoma is a rare type of cancer in salivary glands, most of which are likely to be metastatic. Malignant melanoma of parotid glands occurs almost invariably as metastases from a primary tumor that is located in the skin or the mucous membranes of the head and neck regions.^1^, ^2^, ^3^ There are some case reports regarding metastasizing melanoma to salivary glands from a regressed pigmented skin lesion in head and neck areas.^4^, ^5^ Hence, simultaneously observing a metastatic malignant melanoma in the salivary glands as well as its primary melanoma might not be possible. This paper presented a case of metastatic melanoma with the affected parotid glands who was a 19‐year‐old woman. Moreover, we described her clinical, histological, and immunohistochemical (IHC) features. Accordingly, it was originally from an old nevus on the scalp that must not be neglected and should be detected for ruling out preexisting melanoma. Since every cutaneous pigmented lesion must be pathologically evaluated, in case of any metastatic melanoma in head and neck regions, it is mandatory to search for any previously treated pigmented lesion or the one that has been clinically disappeared.

## 2 CASE PRESENTATION

**FIGURE 1:**
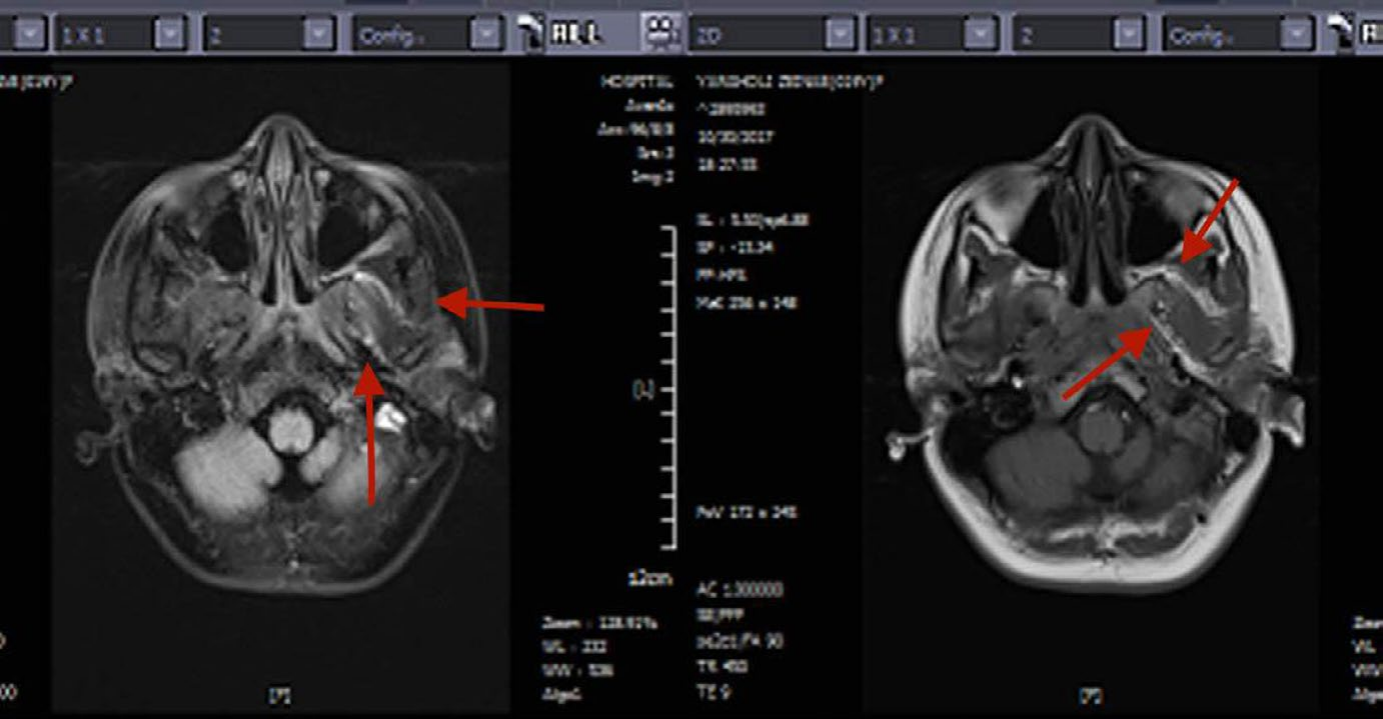
Magnetic resonance imaging (T1 sequence) imaging shows intermediate to high signal intensity in parotid glands

**FIGURE 2:**
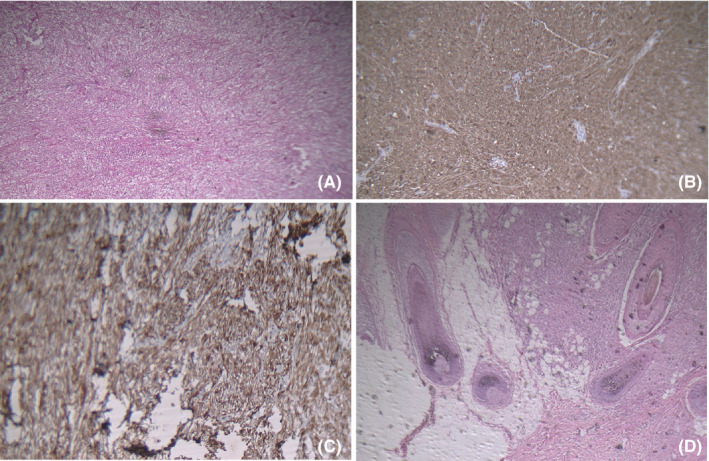
(A) Low power field of section shows spindle cells with storiform pattern. (B) Tumor cells in high power field are HMB45 positive. (C) Tumor cells in low power field show S100 positive. (D) Low power field of skin shows spreading of pigments from epidermis to dermis

A 19‐year‐old woman with pain and swelling in her right parotid gland was referred to Shohada Hospital (Tehran‐Iran) with no underlying diseases. She has complained of a mobile mass on the parotid region for a duration of 3 months. In her clinical examination, the oral mucosa and overlying skin were observed as intact with normal color. The patient's magnetic resonance imaging (MRI) indicated a high signal of a lobular‐contoured, as a 4 × 2.5 cm solid mass lesion in the superior lobe of her right parotid gland (Figure 1). A fine needle aspiration biopsy was performed by a maxillofacial surgeon and then a pathologic report indicated an unknown type of soft tissue malignancy. The primary diagnosis and the surgical treatment plan were discussed for the patient and a written consent was then obtained from her. At the operation session, following conducting the frozen‐sections evaluation, malignant spindle cell tumor was diagnosed. Thereafter, a total parotidectomy was performed. As well, the gross examination of the specimen revealed a solid creamy mass with an almost well‐defined border, invading the superficial lobe of the parotid gland. In addition, the permanent microscopic sections demonstrated a malignant neoplasm composed of fascicles of atypical spindle‐shaped cells with large pleomorphic vesicular nuclei, prominent nucleoli, and clear‐to‐pale eosinophilic cytoplasm arranged in a storiform pattern (Figure 2A). Multinucleated giant cells and lymphocytic infiltration were observed as well. Notably, mitotic activity was about 4/10 HPF. By performing an immunohistochemically analysis, it was found that the atypical cells were HMB45 (Figure 2B) and S100 positive (Figure 2C). However, other markers such as Myo D1, Desmin, PanCK, and CK5/6 were negative. The microscopic feature along with IHC findings was consistent with the result of melanoma diagnosis.

**FIGURE 3:**
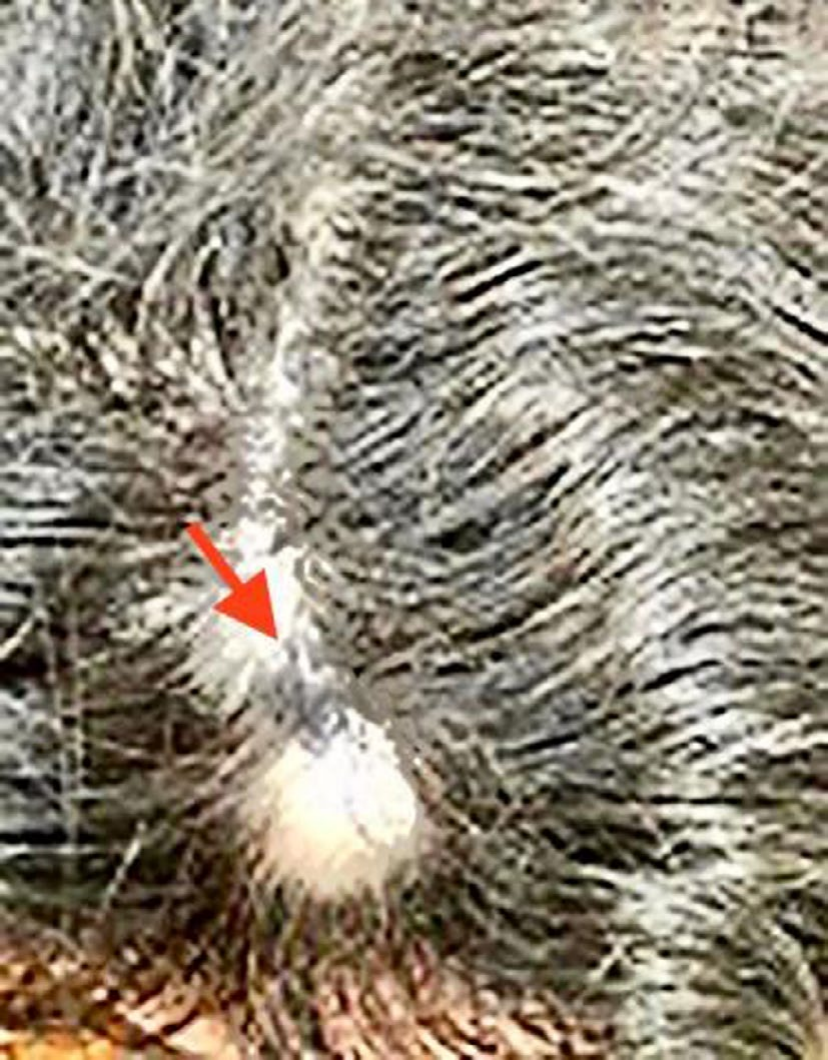
Malignant melanoma on scalp which recurred after 2 years

To discover the primary origin of the recent melanoma lesion, a comprehensive past medical history was taken from the study case. As a result, we found that she had a history of congenital scalp pigmented nevus with no pathological consultation or any report of metastasis after 2 years (Figure 3). Considering the possibility that the scalp lesion may be the origin of metastatic malignant melanoma, we suggested her to undergo excisional biopsy of the newly developed scalp lesion. In this regard, the patient was referred to the dermatology department for both consultation and performing the excisional biopsy operation. Thereafter, the newly formed scalp lesion was surgically removed. As well, the gross examination of the specimen showed safe border margins (0.6–1 cm) as well as a pigmented lesion in the center. Sections also demonstrated skin tissue with dissipated pigments from the epidermis to deep dermis. Moreover, the proliferation of atypical spindle cells scattered in the stroma was arranged from the superficial epidermis to hypodermis with vesicular nuclei and eosinophilic nucleolus (Figure 2D). Of note, the mitotic activity was about 1 in 10 HPF. In this patient, immunohistochemical staining showed the HMB45 intense positivity, but Ki67 was estimated as lower than 5%. Therefore, according to all these findings, the diagnosis of malignant melanoma was confirmed. After a month, she underwent parotidectomy due to swelling and pain in the left parotid gland. The histopathological report showed the same malignant melanoma. Thereafter, adjuvant chemotherapy and radiotherapy were performed for her during 6 and 4 sessions, respectively. She followed her disease by administrating complementary medicines too (homeopathy). After 18 months of follow‐up, the lesion has not recurred and she has still been on a regular follow‐up program.

## 3 DISCUSSION

Melanoma is the most serious skin cancer with a high mortality rate that reminds the necessity of performing the histopathological analysis of any pigmented lesion after biopsy, as well as the detection of the history of pigmented lesions.^6^, ^7^, ^8^ Herein, we presented a rare case of metastatic malignant melanoma originated from a pigmented scalp lesion by passing 2 years from the primary excision of the nevus that had not been examined microscopically before. Moreover, the primary lesion was probably excised with inadequate care regarding the marginal safety in surgical procedure.

Generally, malignant melanomas of the parotid gland are metastatic and have maxillofacial skin origin.^3^ Although around 10–35% of melanomas regress or even completely disappear, some studies demonstrated that malignant melanomas tend to metastasize to lymph nodes and also to parotid glands in some rare occasions.^4^ The parotid tissue is known as an unusual location for metastasis. Since melanocytes are the originated embryo logically from the neural crest and not concerned as a component of salivary tissues, parotid glands seem controversial as the primary origin of melanomas.^3^ Melanoblasts might be considered as a potential source of tumor in the parotid tissue. Accordingly, these might exist in the intralobular duct of this gland.^5^ However, malignant melanoma in salivary glands often is metastatic from a cutaneous primary lesion in both head and neck regions.^1^

According to a research by Greene and Bernier,^9^ melanoblasts would be a substitute of the parotid gland with the down growth of oral epithelium during the development of the parotid. Moreover, they reported that some cells in the normal human parotid gland tested positive for melanin when stained using the L‐DOPA (3,4‐dihydroxy‐L‐phenylalanine) method.^3^ Maier H et al. in their study also reported an amelanotic melanoma of parotid.^10^ Although the existence of primary melanoma of parotid is not impossible, for its definite diagnosis, obtaining accurate medical history of patient is needed. In another study, Kılıçkaya et al. reported a case of malignant melanoma of parotid, the origin of which was detected by earlier photographs of the patient. The photos displayed a pigmented lesion on her ipsilateral face, which spontaneously disappeared.^11^

Addressing the study by Prayson et al., 12 cases were reported who were all diagnosed with malignant melanomas of parotid with cutaneous origins of head and neck areas, except one that had an unknown origin. It is interesting that neither none of them were bilateral nor MRI was used for localizing their tumors.^5^

## 4 CONCLUSION

Melanomas of the head and neck areas can be metastatic to local lymph nodes and parotid glands. Considering the high mortality rate of this malignancy, all pigmented lesions with unusual features, large size, irregular pigmentation, unknown duration, or recent enlargement should be submitted for microscopic examinations. Furthermore, recording the exact past medical history of the affected patients is necessary. In the case of any malignancy, neglecting the marginal safety in the surgical operations of pigmented lesions can increase the probability of the metastasis.

## CONFLICTS OF INTEREST

The authors declare that they have no conflict of interest.

## AUTHOR CONTRIBUTIONS

Author 1: Acquisition of data and diagnosis of the lesion. Author 2: Study supervision and diagnosis of the lesion. Author 3: Initiate the preparation of this case report for scientific publication and surgery of case. Author 4: Study search and design, analysis, and interpretation of data.

## ETHICAL APPROVAL

Shohada_e_Tajrish Educational Hospital, Shahid Beheshti University of Medical Sciences, Oral and Maxillofacial Pathology Department approved this report.

## CONSENT

The authors of this article have obtained all required consent forms from the patient. Her consent has been given for the images and other clinical information to be reported in this article excluding her name.

## Data Availability

Data would be available on request due to privacy/ethical restrictions.
